# Hypertension referrals from community pharmacy to general practice: multivariate logistic regression analysis of 131 419 patients

**DOI:** 10.3399/bjgp18X697925

**Published:** 2018-07-03

**Authors:** Ali Albasri, Suman Prinjha, Richard J McManus, James P Sheppard

**Affiliations:** Nuffield Department of Primary Care Health Sciences, University of Oxford, Oxford.; Nuffield Department of Primary Care Health Sciences, University of Oxford, Oxford.; Nuffield Department of Primary Care Health Sciences, University of Oxford, Oxford.; Nuffield Department of Primary Care Health Sciences, University of Oxford, Oxford.

**Keywords:** general practice, hypertension, logistic regression, new medication, pharmacists, referral

## Abstract

**Background:**

The burden of hypertension in primary care is high, and alternative models of care, such as pharmacist management, have shown promise. However, data describing outcomes from routine consultations between pharmacists and patients with hypertension are lacking.

**Aim:**

To identify factors associated with referral of patients from pharmacies to general practice within the first 2 weeks of starting a new antihypertensive medication.

**Design and setting:**

Multivariate logistic regression conducted on data from community pharmacies in England.

**Method:**

Data were obtained from the New Medicine Service between 2011 and 2012. Analyses were conducted on 131 419 patients. In all, 15 predictors were included in the model, grouped into three categories: patient-reported factors, demographic factors, and medication-related factors.

**Results:**

Mean patient age was 65 years (±13 years), and 85% of patients were of white ethnicity. A total of 5895 (4.5%) patients were referred by a pharmacist to a GP within the first 2 weeks of starting a new antihypertensive medication. Patients reporting side effects (adjusted odds ratio [OR] 11.60, 95% confidence interval [CI] = 10.85 to 12.41) were most likely to be referred. Prescriptions for alpha-blockers were associated with referral (adjusted OR 1.28, 95% CI = 1.12 to 1.47), whereas patients receiving angiotensin-II receptor blockers were less likely to be referred (adjusted OR 0.89, 95% CI = 0.80 to 0.99).

**Conclusion:**

Most patients were followed up by pharmacists without the need for referral. Patient-reported side effects, medication-related concerns, and the medication class prescribed influenced referral. These data are reassuring, in that additional pharmacist involvement does not increase medical workload appreciably, and support further development of pharmacist-led hypertension interventions.

## INTRODUCTION

The New Medicine Service (NMS) is a nationally commissioned NHS contractual service aimed at providing support for patients within the first month of starting a new medication.^1^ It has been delivered in >90% of community pharmacies in England since its introduction in 2011, and covers four therapeutic areas: hypertension, type 2 diabetes, asthma and chronic obstructive pulmonary disease (COPD), and anticoagulation and antiplatelet therapy.^1^ The NMS aims to improve patient adherence to long-term medications by encouraging engagement with their new medication.^1^^,^^2^

Hypertension-related appointments make up almost one in 10 of all GP consultations each year.^3^ With the workload of GPs thought to be nearing saturation point,^4^ alternative models of hypertension management such as pharmacist-led care have the potential to alleviate this increasing burden on primary healthcare systems. Evidence from systematic reviews shows that such interventions can significantly reduce blood pressure compared with usual GP care.^5^^,^^6^

To explore the potential of implementing extended pharmacist roles in the management of hypertension in community settings, detailed assessments of current interventions such as NMS are required to identify patient groups and presentations that pharmacists are able to manage independently, without referral to a GP. Previous research exploring reasons for referral from pharmacies to general practice has focused on upper gastrointestinal symptoms,^7^ and a systematic review of community pharmacy triage services reported reasons for referrals for a variety of other minor ailments.^8^ Much of these data were collected using surveys or questionnaires, relying on participant recall, rather than from routine datasets, and hence may not capture the true impact of such services.

Routinely collected data were analysed from 131 419 patients with hypertension reporting to a community pharmacy with a new antihypertensive medication. This study aimed to establish factors associated with patient referral from pharmacies to general practice within the first 2 weeks of starting a new antihypertensive medication.

## METHOD

### Study design and data source

This was a cross-sectional study of patients with hypertension presenting to a community pharmacy in England with a newly prescribed antihypertensive medication. Anonymised patient and consultation data were recorded by pharmacists using PharmOutcomes software in pharmacies as part of the NMS service, and were subsequently sent to the Pharmaceutical Services Negotiating Committee (PSNC).

The data collection form for pharmacists conducting the NMS can be found in Appendix 1.

**How this fits in**

The burden of hypertension in primary care is high, and alternative models of care, such as pharmacist management, have shown promise. However, data describing outcomes from routine consultations between pharmacists and patients with hypertension are lacking. Within the first 2 weeks of initiating antihypertensive medication, patient referral from pharmacies to general practice were influenced by factors including medication side effects, medication-related uncertainty, and the drug class prescribed. However, overall referral rates were low (4%), suggesting that additional pharmacist involvement does not increase medical workload appreciably, supporting further development of pharmacist-led hypertension interventions.

As this was a secondary analysis of anonymised routinely collected data, no ethical approval was required.

### Participants

Eligible patients were those starting a new medication for either newly diagnosed or pre-existing hypertension. No age, comorbidity, or previous cardiovascular disease restrictions were in place for the NMS. Patients were excluded in this study who:

- withdrew consent for subsequent pharmacist consultations after recruitment;
- could not be contacted for subsequent appointments after enrolling in the service;
- recorded age <18 years, or no age recorded;
- contacted the pharmacist to say that their medication had been stopped before their first NMS consultation; and
- were prescribed the antihypertensive medication for a condition other than hypertension.

### Primary outcome

The primary objective of this study was to establish factors associated with patient referral from community pharmacies to general practice within the first 2 weeks of starting a new antihypertensive medication (intervention NMS stage). The binary outcome of ‘referral’ to general practice was the outcome variable, defined as any instance where ‘referral’ was explicitly marked on the pharmacist-completed NMS form.

### Statistical analysis

Predictors of referral were explored in a multivariate logistic regression model. The lack of previously published literature on predictors of referral from community pharmacies meant that predictor selection was dictated solely by the available data on the NMS form.

Demographic predictors were age, sex, and ethnicity. Medication-related predictors were the antihypertensive drug class newly prescribed. Patient-reported predictors were those included on the NMS form:

- side effects;
- not using the medicine as prescribed;
- negative feelings towards the new blood pressure (BP) medication;
- uncertainty whether the medicine is working;
- not having started the medication since being prescribed;
- concerns with remembering to take the medication;
- difficulty with the medication’s formulation;
- prescriber stopping the medication;
- more information requested about the new medication;
- a missed dose in the past 7 days; and
- using the medicine as prescribed.

Given the small number of predictors and large number of outcome cases, no attempt was made to reduce the model through predictor selection.

Calcium channel blockers (CCBs) were selected as the reference medication class against which other medication classes were compared, because they were the most commonly prescribed drug class in the dataset, and the average sample population age was >55 years.^9^ White ethnicity was chosen as the reference standard against which all other ethnicities were measured. Age categories were split as follows: <40 years (comparator category), 40–49 years, 50–59 years, 60–69 years, 70–79 years, and ≥80 years. All other predictors were binary variables (for example, side effects reported: yes or no).

Results are presented as odds ratios (OR) and associated 95% confidence intervals (CIs). Further analyses examined interactions between age and side effects, and sex and side effects.^10^

## RESULTS

### Inclusion of participants

Anonymised data from 148 412 patients with hypertension were available; collected from 1 October 2011 to 30 September 2012, of whom 131 419 (88.6%) matched the authors’ inclusion criteria. A flow chart of excluded patients can be found in Appendix 2. The characteristics of the excluded participants are shown in Appendix 3. No systematic differences were observed between the excluded patients and the analysed sample.

### Descriptive data

Patients attended pharmacies based in all nine regions of England. Mean patient age was 65 (±13) years. The majority of patients were of white ethnicity (85%), with the rest South Asian (4%), black/African-Caribbean (2%), ‘not stated’ (8%), or ‘other’ (1%) (Table 1).

**Table 1.** Dataset characteristics

**Total population, *n***	**131 419**

**Mean age, years (SD)**	65 (13)

**Ethnicity, *n* (%)**	
White	112 194 (85)
South Asian	4586 (4)
Black/African-Caribbean	2760 (2)
Not stated	10 234 (8)
Other	1645 (1)

**Sex, *n* (%)**	
Male	61 146 (47)
Female	70 273 (53)

**Medication class, *n* (%)**	
CCB	41 372 (32)
ACEi	34 497 (26)
ARB	17 415 (13)
Beta-blocker	16 211 (12)
Thiazide diuretic	15 487 (12)
Alpha-blocker	5264 (4)
Other	1173 (1)

**Proportion of pharmacies, by location, contributing to dataset, %**	
North East	6
North West	16
Yorkshire and the Humber	10
East Midlands	9
West Midlands	10
East Anglia	5
Greater London	15
South East	15
South West	14

*ACEi* = *angiotensin-converting enzyme inhibitor. ARB* = *angiotensin II receptor blocker. CCB* = *calcium channel blocker.*

The proportions of BP-lowering medications prescribed closely reflect the recommended order of prescribing in the current National Institute for Health and Care Excellence (NICE) guidelines, 2011;^9^ with CCBs most common (41 372, 32%), followed by angiotensin-converting enzyme inhibitors (ACEi, 34 497, 26%), angiotensin II receptor blockers (ARBs, 17 415, 13%), beta adrenoceptor blockers (beta-blockers, 16 211, 12%), thiazide diuretics (15 487, 12%), alpha adrenoceptor blockers (alpha-blockers, 5264, 4%), and ‘other antihypertensives’, such as centrally acting antihypertensives (1173, 1%).

Side effects were reported by 24 992 (19%) patients, while 107 989 (82.2%) reported that they used their new antihypertensive medication as prescribed. Table 2 shows the frequencies of all predictor variables.

### Primary outcome

**Figure 1.**
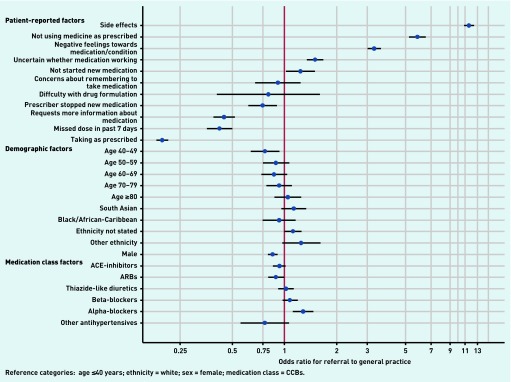
***Coefficient plot showing adjusted odds ratios for referral to general practice for included predictors. ACE* = *angiotensin-converting enzyme. ARBs* = *angiotensin II receptor blockers. CCBs* = *calcium channel blockers.***

A total of 5895 patients (4.5%) were referred back by a pharmacist to a GP within the first 2 weeks of starting a new antihypertensive medication (Table 2). Patients reporting side effects from their new medication were most likely to be referred to their GP (adjusted OR 11.60, 95% CI = 10.85 to 12.41) (Figure 1). Other patient-reported factors, such as expressing negative feelings towards the antihypertensive drug (adjusted OR 3.29, 95% CI = 3.01 to 3.60), and reporting uncertainty regarding the efficacy of the new medication (adjusted OR 1.50, 95% CI = 1.35 to 1.68), were significantly associated with referral. In contrast, patients who were provided with more information about their new medication from the pharmacist (adjusted OR 0.45, 95% CI = 0.39 to 0.52), and those using their medications as prescribed (adjusted OR 0.20, 95% CI = 0.18 to 0.21), were less likely to be referred to their GP.

**Table 2.** Logistic regression of factors associated with referral from pharmacy to general practice for 131 419 patients

	***n* (%)**	**Referred *n* (%)**	**Adjusted OR (95% CI)**
**Outcome variable**			

Referrals from pharmacy to general practice	5895 (4.5)	–	–

**Predictor variables**			

**Patient-reported factors**			
Side effects	24 992 (19.0)	4155 (16.6)	11.60 (10.85 to 12.41)
Not using the medicine as prescribed	5271 (4.0)	2236 (42.4)	5.83 (5.23 to 6.52)
Negative feelings towards new BP medication	5038 (3.8)	1292 (25.6)	3.29 (3.01 to 3.60)
Uncertainty whether medicine working	8344 (6)	555 (6.7)	1.50 (1.35 to 1.68)
Not yet started the medication	823 (0.6)	230 (27.9)	1.23 (1.02 to 1.50)
Concerns with remembering to take medication	965 (0.7)	60 (6.2)	0.91 (0.68 to 1.24)
Difficulty with medication formulation	149 (0.1)	13 (8.7)	0.81 (0.41 to 1.60)
Prescriber stopping the medication	777 (0.6)	313 (40.3)	0.75 (0.62 to 0.91)
More information about medication needed	7276 (5.5)	301 (4.1)	0.45 (0.39 to 0.52)
Missed dose in the past 7 days	1166 (0.9)	415 (35.6)	0.42 (0.36 to 0.50)
Using medicine as prescribed	107 989 (82.2)	2354 (2.2)	0.20 (0.18 to 0.21)

**Demographic factors**			

**Age, years (**<**40 years as reference)**			
40–49	12 987 (9.9)	463 (3.6)	0.77 (0.63 to 0.93)
50–59	23 451 (17.8)	951 (4.1)	0.89 (0.75 to 1.06)
60–69	37 312 (28.4)	1548 (4.1)	0.87 (0.74 to 1.03)
70–79	34 895 (26.6)	1680 (4.8)	0.93 (0.78 to 1.10)
≥ 80	17 975 (13.8)	1045 (5.8)	1.05 (0.88 to 1.25)

**Ethnicity (white as reference)**			
South Asian	4586 (3.5)	227 (4.9)	1.14 (0.97 to 1.35)
Other	1645 (1.3)	91 (5.5)	1.25 (0.97 to 1.62)
Not stated	10 234 (7.8)	476 (4.7)	1.12 (1.00 to 1.26)
Black/African-Caribbean	2760 (2.1)	127 (4.6)	0.94 (0.75 to 1.17)

**Sex, male (female as reference)**	61 146 (46.5)	2172 (3.6)	0.85 (0.80 to 0.91)

**Medication-related factors (CCB as reference)**			
Alpha-blocker	5264 (4.0)	411 (7.8)	1.28 (1.12 to 1.47)
Beta-blocker	16 211 (12.3)	777 (4.8)	1.10 (0.99 to 1.21)
Thiazide	15 487 (11.8)	800 (5.2)	1.02 (0.92 to 1.13)
ACEi	34 497 (26.2)	1312 (3.8)	0.94 (0.86 to 1.02)
ARB	17 415 (13.3)	622 (3.6)	0.89 (0.80 to 0.99)
Other	1173 (0.9)	53 (4.5)	0.77 (0.56 to 1.06)

*ACEi* = *angiotensin-converting enzyme inhibitor. ARB* = *angiotensin II receptor blocker. BP = blood pressure. CCB* = *calcium channel blocker. OR* = *odds ratio.*

Neither ethnicity nor age were significant predictors of referral from pharmacy to general practice. However, males were less likely to be referred than females (adjusted OR 0.85, 95% CI = 0.80 to 0.91).

Alpha-blockers were associated with referral (adjusted OR 1.28, 95% CI = 1.12 to 1.47), whereas patients prescribed an ARB were significantly less likely to be referred (adjusted OR 0.89, 95% CI = 0.80 to 0.99). No other drug classes were associated with referral.

### Interaction analyses

No interaction was found between age and side effects (OR 1.01, 95% CI = 1.00 to 1.01), meaning that the likelihood of referral was not significantly affected by the reporting of side effects as age increased. However, males reporting medication side effects were more likely to be referred (OR 1.19, 95% CI = 1.06 to 1.34) than females reporting side effects.

Further post-hoc analysis showed that patients who reported side effects and were offered additional information about their medication or condition were less likely to be referred to their GP than those reporting side effects without receiving additional information (OR 0.42, 95% CI = 0.32 to 0.54).

## DISCUSSION

### Summary

The results indicate that within the first 2 weeks of starting a new antihypertensive medication the majority of patients with hypertension (95.5%) were supported without further recourse to their GP. The study highlights several important factors associated with patient referral to general practice. Side effects, uncertainty regarding the efficacy of the medication, and negative feelings towards the newly prescribed antihypertensive drug were among the patient-reported factors most associated with re-referral. The antihypertensive drug class most associated with referral was alpha-blockers, while ARBs were least associated with referral. Other potentially important factors, such as BP levels, comorbidities, and polypharmacy, were not routinely collected in the dataset, however, and could have confounded results.

### Strengths and limitations

To the authors’ knowledge, this is the first study exploring factors associated with referral from pharmacies to general practice in patients newly prescribed an antihypertensive medication. The large sample size and nationally representative coverage of the data make this analysis an important addition to the evidence base. Although previous studies assessing referral from pharmacies to general practice have used survey data,^8^ the authors were able to access and use, to their knowledge, the only routinely collected dataset from a nationally commissioned service for hypertension in English pharmacies.

The full range of factors associated with referral from pharmacies to general practice may be limited by the lack of data on patient comorbidities, the number of antihypertensive medications prescribed to each patient, and other prescribed medications. Previous studies have found that polypharmacy is associated with non-adherence to antihypertensive medications,^11^ and hence possible increases in GP visits. Furthermore, the omission of routinely recorded BP readings within the consultations means that this potentially important predictor for referral was not present in this analysis. In addition, reasons for referral such as drug interactions, although present on the NMS form, were not present in the dataset. Although information regarding drug interactions was discussed with 9538 (7%) patients, the incomplete data regarding the attribution of referral to active or potential drug interactions prevented further analysis.

An inherent limitation within the delivery of the NMS is the potential for pharmacists to select patients who are more engaged with their condition, or for more engaged patients to accept the service. Information on patients who rejected the service would have strengthened the findings of these data and would allow the generalisability of the results to be established more clearly.

### Comparison with existing literature

A survey of 1782 patients with hypertension suggested that patient-reported problems, such as difficulty accepting their diagnosis or frustration with treatment, were associated with higher levels of self-reported medication non-compliance.^12^ These data, in line with the authors’ results showing the relationship between patient-reported problems and referral to general practice, further highlight the importance of addressing patient-reported problems in the management of patients with hypertension. Knowledge of these problems from this analysis is a useful addition to future blood pressure-related consultations between clinicians and patients.

ARBs were the drug class least associated with referral (adjusted OR 0.89, 95% CI = 0.80 to 0.99). This, in part, could be due to the lower association with adverse events over other antihypertensive drug classes, as evidenced in a systematic review by Thomopoulos *et al*.^13^ Their meta-analysis found that ARBs were the only antihypertensive drug class that does not significantly increase adverse event rates over placebo (risk ratio 1.13, 95% CI = 0.78 to 1.62).

Alpha-blockers were the drug class most associated with referral to GP (adjusted OR 1.28, 95% CI = 1.12 to 1.47). Alpha-blockers are often the fourth antihypertensive medication added to a patient’s regimen, tending to be reserved for patients with resistant hypertension.^9^ They can cause pronounced postural hypotension and dizziness, particularly within the first few days of initiating. Thomopoulos *et al* showed that the incidence of BP medication discontinuation due to adverse events is proportional to the number of antihypertensive medications a patient takes. Therefore the association between alpha-blockers and referral could be due to their side effect profile, or due to the fact that they serve as a proxy for patients taking multiple BP-lowering medications.

A systematic review of 18 trials (4168 patients) indicated that the time taken to reach 50% of the maximum estimated BP-lowering effect across antihypertensive drug classes was 1 week.^14^ This suggests that pharmacists should not be routinely referring patients back to their GP due to lack of drug efficacy after 1–2 weeks, as was observed in this dataset. Given that the clinical interpretation of BP readings taken in community pharmacies is less well defined in comparison to home readings,^15^ the most appropriate source of BP results on which to base this decision should be carefully considered.^16^^,^^17^

### Implications for research and practice

Patients reporting side effects from their new medication were the most likely to be referred to their GP in this cohort of 131 419 patients (adjusted OR 11.60, 95% CI = 10.85 to 12.41). The likely responses from clinicians to the development of such side effects could include dose reductions or switching to alternative medications. Pharmacists are highly trained in the use and management of medications, and so these changes are within their professional capacity. An extended prescribing role for community pharmacists could reduce referral of routine cases to an already overstretched general practice, but would require many to undertake further training. Alternatively, efficient communication systems between pharmacists and GPs could result in reduced patient referrals by allowing pharmacists to implement changes to medications or doses in a more timely manner in pharmacies.^18^ Digital interventions involving the telemonitoring of blood pressure coordinated by pharmacists have shown promise,^19^ and could serve as a useful tool for pharmacists to deliver such changes.

The reporting of negative feelings towards the new medication and uncertainty as to their efficacy were also independent predictors of referral. These concerns could be managed via BP measurements, verbal advice, and reassurance, and do not require a pharmacist to have a prescribing qualification. Their significance in the results suggests that patients may not be accepting pharmacist advice and recommendations, or that pharmacists may not consistently offer this.

The impact on clinical workload of additional pharmacy services is worthy of consideration. With the incidence of new hypertension cases at eight people per 1000,^20^ a 4% referral rate would mean that of the 52 new hypertension cases per practice per year^21^ only two would need to be seen again by their GP within 2 weeks of medication initiation. Without knowing the rate of patient re-presentation to GPs outside of the NMS, it is difficult to determine whether a 4% referral rate would indeed represent an improvement or worsening of the current situation; however, these numbers appear low.

Systematic evaluations are now required to quantify the clinical benefit of such programmes over usual care before development of new or extended hypertension services that involve the clinical follow-up of patients in pharmacies.

Future research should aim to analyse clinical notes made by pharmacists following consultations with patients with hypertension, making clear the specific advice or recommendations pharmacists are providing.

## Appendix 3. Characteristics of excluded patients

**Table:**

**Population**	16 993

**Age (mean, SD)**	N/A

**Sex, *n* (%)**	
Male	7580 (45)
Female	9413 (55)

**Medication class, *n* (%)**	
CCB	5312 (31)
ACEi	4396 (26)
ARB	1816 (11)
Thiazide	2147 (13)
Beta-blocker	2342 (14)
Alpha-blocker	799 (5)
Other	181 (1)

**Ethnicity, *n* (%)**	
White	14 163 (83)
South Asian	479 (3)
Black/African-Caribbean	360 (2)
Not stated	1736 (10)
Other	255 (2)

*ACEi* = *angiotensin-converting enzyme inhibitor. ARB* = *angiotensin II receptor blocker. CCB* = *calcium channel blocker.*
